# Effects of an 8-Week Protein Supplementation Regimen with Hyperimmunized Cow Milk on Exercise-Induced Organ Damage and Inflammation in Male Runners: A Randomized, Placebo Controlled, Cross-Over Study

**DOI:** 10.3390/biomedicines8030051

**Published:** 2020-03-04

**Authors:** Sihui Ma, Takaki Tominaga, Kazue Kanda, Kaoru Sugama, Chiaki Omae, Shunsuke Hashimoto, Katsuhiko Aoyama, Yasunobu Yoshikai, Katsuhiko Suzuki

**Affiliations:** 1 Faculty of Sport Sciences, Waseda University, Tokorozawa 359-1192, Japan; masihui@toki.waseda.jp; 2 Graduate School of Sport Sciences, Waseda University, Tokorozawa 359-1192, Japan; t.tominaga7713@gmail.com (T.T.); chappy-ld312@moegi.waseda.jp (C.O.); 3 Research Institute for Life Support Innovation, Waseda University, Shinjuku 162-0041, Japan; rurishijimikanda@gmail.com (K.K.); k.sugama@kurenai.waseda.jp (K.S.); 4 Ortho Corporation, Shibuya 150-0002, Japan; hashimoto@kenko.co.jp (S.H.); aoyama@kenko.co.jp (K.A.); 5 Division of Host Defense, Medical Institute of Bioregulation, Kyushu University, Fukuoka 812-8582, Japan; yoshikai@bioreg.kyushu-u.ac.jp

**Keywords:** hyperimmunized milk, exercise, inflammation, intestinal permeability, cytokine

## Abstract

Prolonged strenuous exercise may induce inflammation, cause changes in gastrointestinal permeability, and lead to other unfavorable biological changes and diseases. Nutritional approaches have been used to prevent exercise-induced inflammatory responses and gastrointestinal disorders. Hyperimmunized milk, obtained by immunizing cows against specific antigens, promotes the development of immunity against pathogens, promotes anti-inflammatory effects, and protects intestinal function. Immune protein (IMP) is a concentrated product of hyperimmunized milk and is a more promising means of supplementation to protect against acute infections and inflammation. To determine whether IMP has protective properties against exercise-induced gastrointestinal dysfunction and inflammation, we examined biochemical markers, intestinal damage markers, and pro-/anti-inflammatory profiles of young male runners using a randomized, placebo controlled, cross-over design. Urine samples were collected and used for measurements of creatinine, *N*-acetyl-β-d-glucosaminidase, osmotic pressure, and specific gravity. Titin was measured as a muscle damage marker. Further, urine concentrations of complement 5a, calprotectin, fractalkine, myeloperoxidase, macrophage colony-stimulating factor, monocyte chemotactic protein-1, intestinal fatty acid binding protein (I-FABP), interferon (IFN)-γ, interleukin (IL)-1β, IL-1 receptor antagonist, IL-2, IL-4, IL-6, IL-8, IL-10, IL-12p40, and tumor necrosis factor (TNF)-α were measured by enzyme-linked immunosorbent assays. We demonstrated that urine osmotic pressure, urine specific gravity, I-FABP, IFN-γ, IL-1β, and TNF-α were reduced by 8 weeks of IMP supplementation, indicating that IMP may have potential in preventing strenuous exercise-induced renal dysfunction, increased intestinal permeability, and inflammation. Thus, IMP supplementation may be a feasible nutritional approach for the prevention of unfavorable exercise-induced symptoms.

## 1. Introduction

Prolonged strenuous exercise may induce unfavorable biological changes and symptoms, including inflammatory responses, such as leukocyte infiltration [1,2,3]; gastrointestinal (GI) incidents, such as diarrhea, nausea, and gastric pain [4,5,6]; delayed-onset muscle soreness; muscle and internal organ injury; and immune suppression [7,8,9,10,11].

Strenuous exercise can also induce intestinal barrier dysfunction. The GI mucosa serves as the first line of defense against invasion from non-self antigens [12]. Functional loss of the GI barrier, consisting of the enterocyte membranes, tight junctions, mucous, and localized macrophages, may bring unwanted biological and pathological consequences by allowing harmful substances (e.g., bacteria, xenobiotics, hydrolytic enzymes, and so forth) to enter into the circulation [13].

Many athletes use aspirin, ibuprofen, and other non-steroidal anti-inflammatory drugs (NSAIDs) to treat inflammation-induced algesthesia [14]. However, NSAIDs inhibit cyclooxygenase (COX) in the GI mucosa, aggravating the GI symptoms and harming athletes’ performance and wellbeing. Therefore, supplementation for protecting the GI barrier merits consideration [15].

Hyperimmunized milk is obtained by immunizing cows against specific antigens. This technique results in the enrichment of various immunoglobulins in the milk product [16]. In 1957, Stolle first produced hyperimmunized milk by injecting cows with various bacteria pathogenic to humans (26 kinds of antigens, including *Escherichia coli*, *Salmonella typhimurium*, *Shigella dysenteriae*, *Staphylococcus pyogenes*, *Proteus vulgaris*, and others) [16]. Hyperimmunized milk is reported to possess health-promoting effects, including immunoregulatory, performance-enhancing effects, and NSAID-induced GI damage-preventing effects [17,18,19,20]. For instance, oral administration of milk from cows hyperimmunized against pathogenic bacteria is reported to down-regulate inflammatory responses in the gut, and may reduce allergic disease [21], protect against radiation-induced opportunistic infection and lethality [22,23], alleviate inflammatory responses to carrageenin [24], abrogate the lymphocyte response to concanavalin A [25], and prevent or treat neonatal bacterial infection [26].

Hyperimmunized protein, also known as “immune protein (IMP)”, is a concentrated product of hyperimmunized milk, and therefore provides more promise as a supplement to protect individuals from acute inflammation. Wang et al. reported that oral administration of bovine milk from hyperimmunized cows down-regulated Th17 and Th2 responses and reduced interleukin (IL)-17A in the gut [21]. Additionally, IL-1β, IL-2, IL-6, and tumor necrosis factor (TNF)-α production were reduced, and collagen-induced arthritis was reported as being alleviated with 49 days of administration of hyperimmune colostrum [27]. When injected with a novel anti-inflammatory factor isolated from milk from hyperimmunized cows, a mastitis mouse model showed less mammary inflammation, and edema was suppressed by as much as 80% in carrageenin-challenged rats [24]. However, to the best of our knowledge, the protective effect of neither hyperimmunized milk nor IMP on exercise-induced inflammation and other adverse events have been reported to date. Therefore, we sought to test the potential benefits of IMP supplementation on prolonged strenuous exercise.

In order to determine whether IMP has protective properties on exercise-induced organ damage and inflammation, we examined biochemical markers, cytokine excretion profiles, and organ damage markers in young male runners undergoing a 3000 m full-speed running test (3000 m time trial (3000 m TT)) with or without 8-week IMP supplementation.

## 2. Materials and Methods

### 2.1. Experimental Design

**Figure 1:**
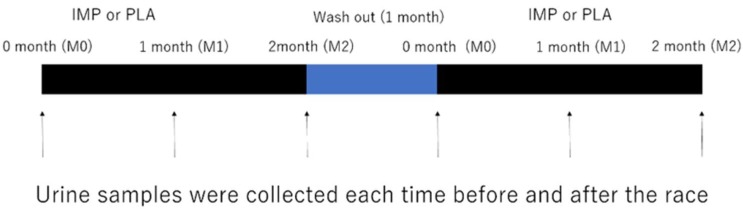
Experimental design. Immune protein powder (IMP) or a matched placebo (normal protein powder, PLA) were administered for 2 months in a cross-over randomized controlled trial (RCT). Arrows indicate the times at which 3000 m time trials were performed.

We designed a double-blind randomized cross-over placebo-controlled study. The participants were recruited to participate in two separate experimental trials with a 1 month wash out period: supplementation of either (1) immune protein (IMP trial) or (2) placebo protein (PLA trial). All of the participants were asked to complete three separate 3000 m TT at three time points in each trial (first race: before supplementation, M0; second race: 1 month after supplementation, M1; third race: 2 months after supplementation, M2) (Figure 1). The experimental protocols were approved by the Ethics Committee of Waseda University (2017-319, date of approval: 11 April 2018). Written informed consent was obtained from all the participants prior to their enrollment in the study. The experiments were carried out from April 2018 to January 2019.

### 2.2. Participants

Seven young men participated in this study. The participants were recreationally active and had no chronic diseases. The characteristics of the participants were as follows: age, 18.7 ± 1.5 (mean ± standard deviation, SD) years; height, 171.8 ± 7.7 cm; body mass, 60.4 ± 3.1 kg. Participants read and signed an informed consent form prior to engaging in the study. Inclusion criteria were as follows: (a) participants had to be male long-distance runners from Waseda University between the ages of 18 and 30 years old; (b) participants had to be healthy and free of any known disease, determined by a medical history questionnaire; (c) participants had to be individuals who do not change training loads and diet content/amount during the experimental period; and (d) participants had to abstain from supplemental protein or amino acids for 3 months prior to participating. A physical activity questionnaire and medical history form were filled out prior to participation to establish that physical activity requirements were met and to identify potential risk factors that could be aggravated by participation in the study.

### 2.3. Immune Protein Supplementation

Following an initial baseline 3000 m TT, subjects were asked to take IMP powder (IMP trial) or a matched placebo (normal protein powder, PLA trial) two times a day for 8 weeks. IMP and placebo were provided in packaged, single doses (10 g), and were obtained from Ortho Inc. (Ortho Incorporation, Tokyo, Japan). IMP and placebo are administrated by mixing with water.

### 2.4. Experimental Protocol

After recruitment, the subjects were counter-balanced on the basis of body weight and 3000 m TT into a PLA or IMP trial. There were no significant differences between groups for any of these variables.

The subjects carried out 3000 m TTs at time point of M0, M1, and M2. The time trials were carried out on an athletic field. The food and fluid intake of subjects was not restricted on the days of the races (Figure 1). IMP or PLA were consumed over 2 months, separated by a 1-month wash-out period, according to previous studies [28,29,30]. Ratings of perceived exertion (RPE) were obtained using the Borg scale. Body composition and body fat percentage were obtained using an InBody 720 Body Composition Analyzer (Inbody Japan Inc., Tokyo, Japan). Participants were asked to self-report if they experienced discomfort during the experiment period.

### 2.5. Urine Sampling and Analysis

Pre-exercise urine samples (Pre) were collected 30–60 min before each 3000 m TT. After the pre-sampling, participants were allowed to perform warm-up exercises ad libitum prior to the 3000 m TT. Post-exercise urine samples (Post) were collected within 30 min after completion of the time trial. The urinary excretion of participants was not restricted before exercise. The collected urine samples were centrifuged at 1000× *g* for 10 min to remove sediments. The supernatants were stored at −80°C until analysis.

### 2.6. Assays for Urine Biochemistry, Inflammatory Substances, and Organ Damage Markers

Urine creatinine, *N*-acetyl-β-d-glucosaminidase (NAG), osmotic pressure, and specific gravity were measured by Koutou-Biken Co. (Koutou-Biken Co., Tsukuba, Japan).

IL-1β, IL-6, and TNF-α concentrations were measured with Quantikine high sensitivity (HS) enzyme-linked immunosorbent assay (ELISA) kits (R&D Systems Inc., Minneapolis, MN, USA). IL-1 receptor antagonist (ra), monocyte chemotactic protein (MCP)-1, and macrophage colony-stimulating factor (M-CSF) concentrations were also measured with Quantikine ELISA kits (R&D Systems Inc., Minneapolis, MN, USA). Intestinal fatty acid binding protein (I-FABP) and fractalkine concentrations were measured with Duoset ELISA kits (R&D Systems Inc., Minneapolis, MN, USA). IL-2, IL-4, IL-8, IL-10, IL-12p40, complement 5a (C5a), and interferon (IFN)-γ concentrations were measured with OptEIA ELISA kits (Becton Dickinson Biosciences, San Diego, CA, USA). Calprotectin and myeloperoxidase (MPO) concentrations were measured with ELISA kits (Hycult biotechnology Inc, Uden, The Netherlands). Titin concentration was measured by ELISA as previously described [31]. The absorbance was measured spectrophotometrically on a VersaMax Microplate Reader (Molecular Devices Inc., San Jose, CA, USA) according to the manufacturer’s instructions. The concentration of each protein was calculated by comparison with a calibration curve established in the same measurement.

### 2.7. Statistical Analysis

The sample size was calculated using the program G*Power [32]. Six subjects were required to detect an effect size of *f* = 0.5 for the within-between interaction, with a power of 0.8 and a significance level of 0.05 under the assumption of a correlation coefficient among repeated measures *r* = 0.7 and a nonsphericity correction of *ε* = 0.5. Data are presented as means ± SD. A two-way analysis of variance (ANOVA) was performed to determine the main effects of protein (IMP or PLA) or time points for 3000 m race time, body composition, ΔRPE, and body fat percentage. A three-way ANOVA with mixed-effects analysis was performed to determine the main effects of protein (IMP or PLA), time points (M0, M1, and M2), and exercise. Urine creatinine was used for urinary parameters’ correction. Statistical significance was defined as *p* < 0.05. Statistical analysis was performed using GraphPad 8.0 (Graphpad, Ltd., La Jolla, CA, USA).

## 3. Results

### 3.1. Body Composition, RPE, and 3000 m Time Trial Results

**Figure 2:**
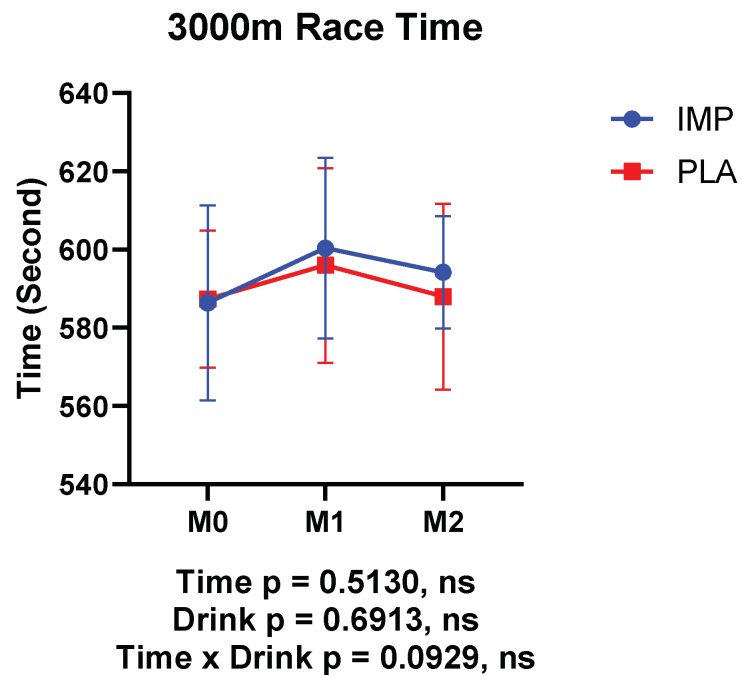
Results of 3000 m time trials. The data are presented as means ± SD.

**Figure 3:**
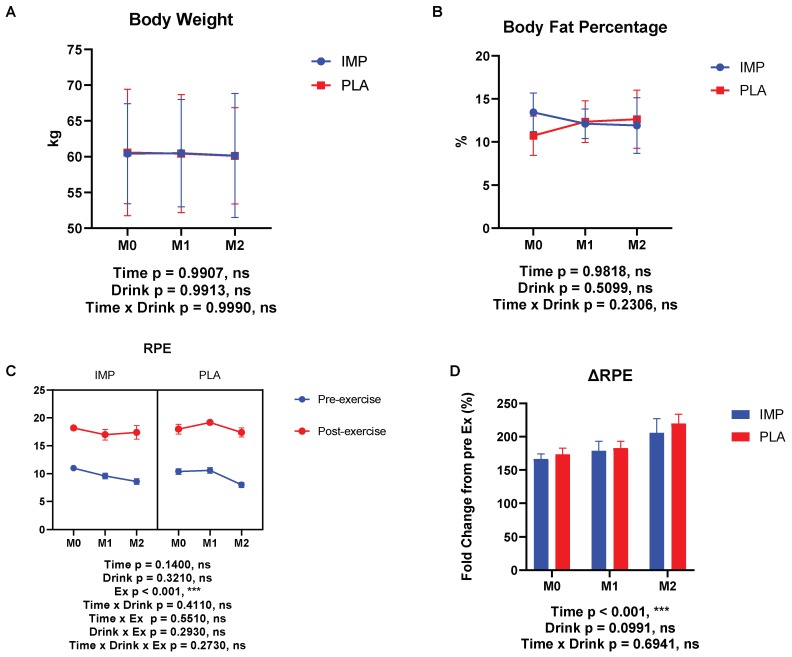
Body composition, body fat percentage, and ratings of perceived exertion (RPE) results. (**A**) Body weight change, (**B**) change in body fat percentage, (**C**) RPE at each time point, and (**D**) RPE change between IMP and PLA. The data are presented as means ± SD. *** p < 0.001.

No significant interaction was observed between IMP and PLA in race time (Figure 2). Body weight, body fat percentage, and RPE were not different between participants in the IMP trial and PLA trial (Figure 3). No adverse events were self-reported or observed during the study period.

### 3.2. Renal Function Markers

**Figure 4:**
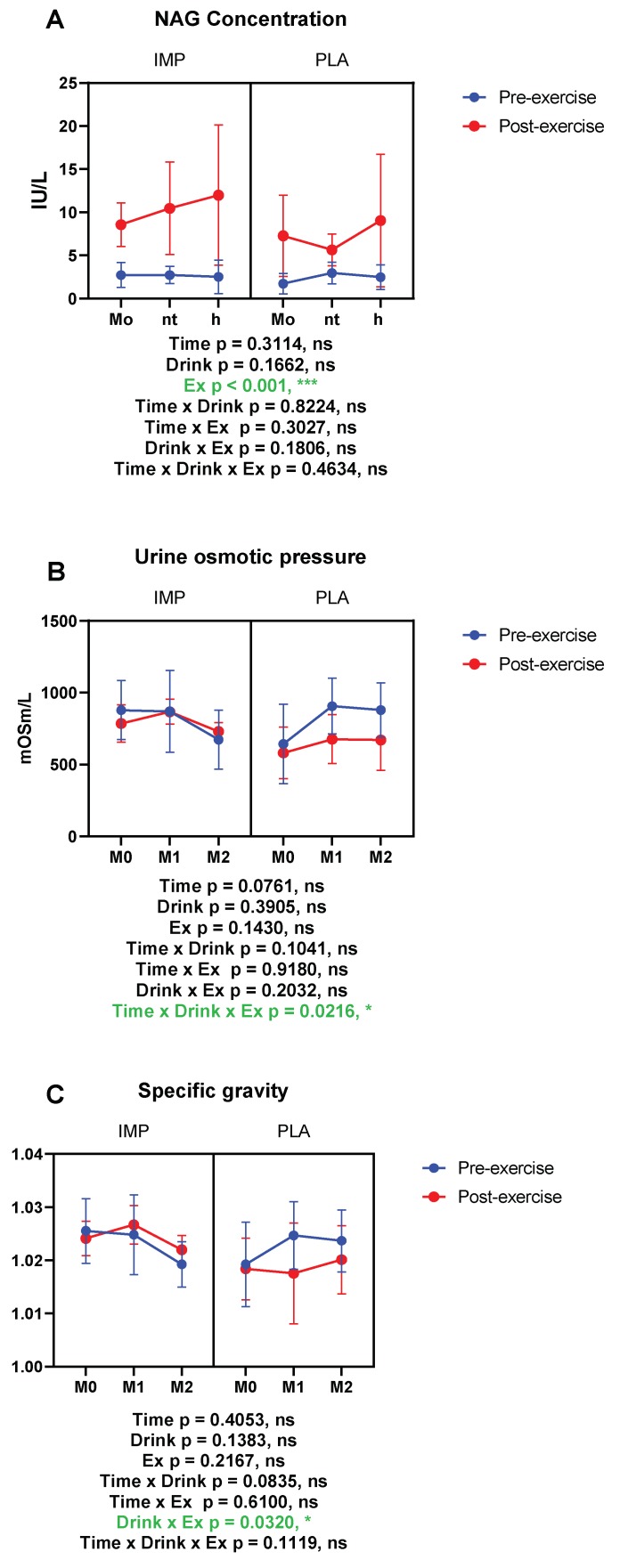
Results of renal function. (**A**) *N*-acetyl-β-d-glucosaminidase (NAG) concentration, (**B**) urine osmotic pressure, and (**C**) urine-specific gravity. The data are presented as means ± SD. * p < 0.05, and *** p < 0.001. Green text indicates a significance <0.05

NAG, urine osmotic pressure, and specific gravity were measured as indicators of renal function. The right side of each figure in Figure 3 shows the data of the PLA supplementation period and the left side shows the data of the IMP supplementation period. Urine NAG was increased by exercise, indicating renal damage occurred after 3000 m TT (Figure 4A). However, in the IMP period, significant interaction effects on urine osmotic pressure changed and specific gravity change were observed (Figure 4B,C). Summary data are available in Table 1. 

### 3.3. Intestine Damage Marker

**Figure 5:**
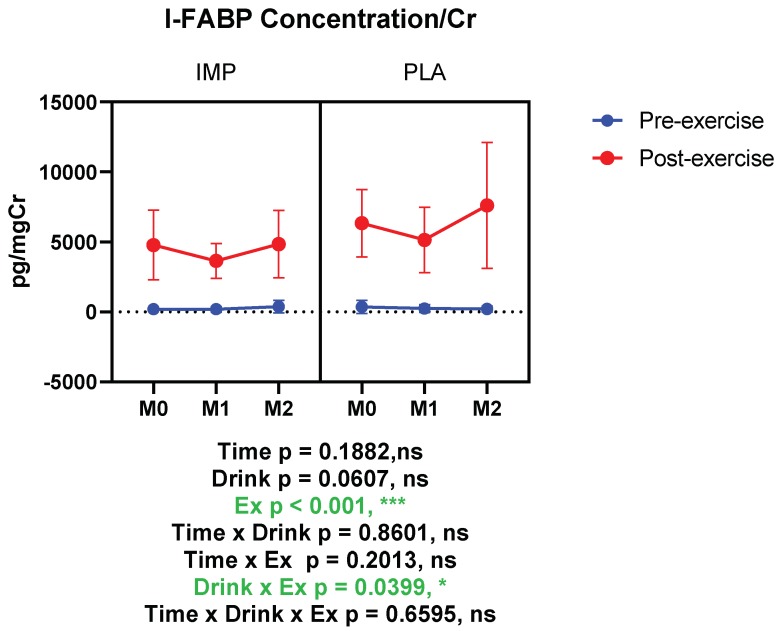
Urine intestinal fatty acid binding protein (I-FABP) concentration corrected with creatinine. The data are presented as means ± SD. * p < 0.05 and *** p < 0.001. Green text indicates a significance <0.05.

I-FABP was measured as an intestinal damage marker. According to our results, I-FABP was increased significantly after 3000 m TTs, indicating intestinal damage. When I-FABP concentration was corrected with creatinine, it indicated that IMP supplementation attenuated the exercise-induced increase of I-FABP significantly (Figure 5). Summary data are available in Table 1. 

### 3.4. Muscle Damage Marker

**Figure 6:**
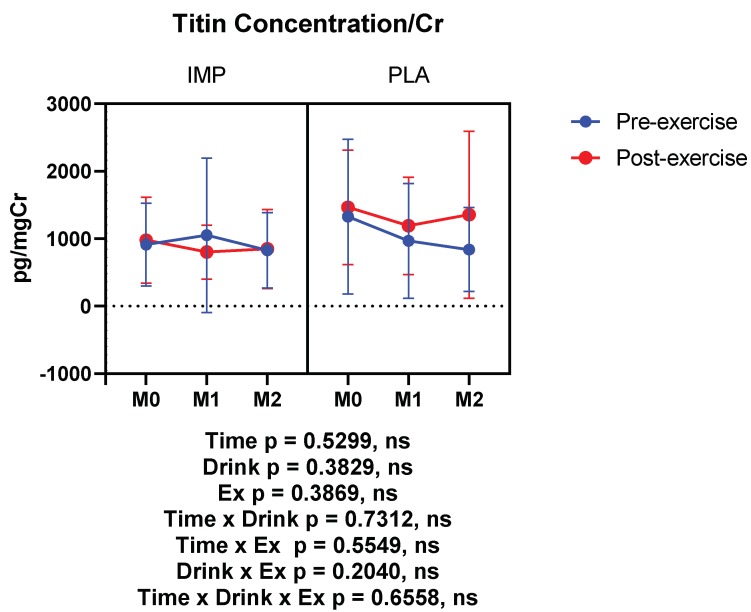
Urine titin concentration corrected with creatinine. The data are presented as means ± SD.

Titin was measured as a muscle damage marker, which was not altered by 3000 m TTs. IMP did not alter titin concentration (Figure 6). Summary data are available in Table 1. 

### 3.5. Inflammatory Substance Profile

**Figure 7:**
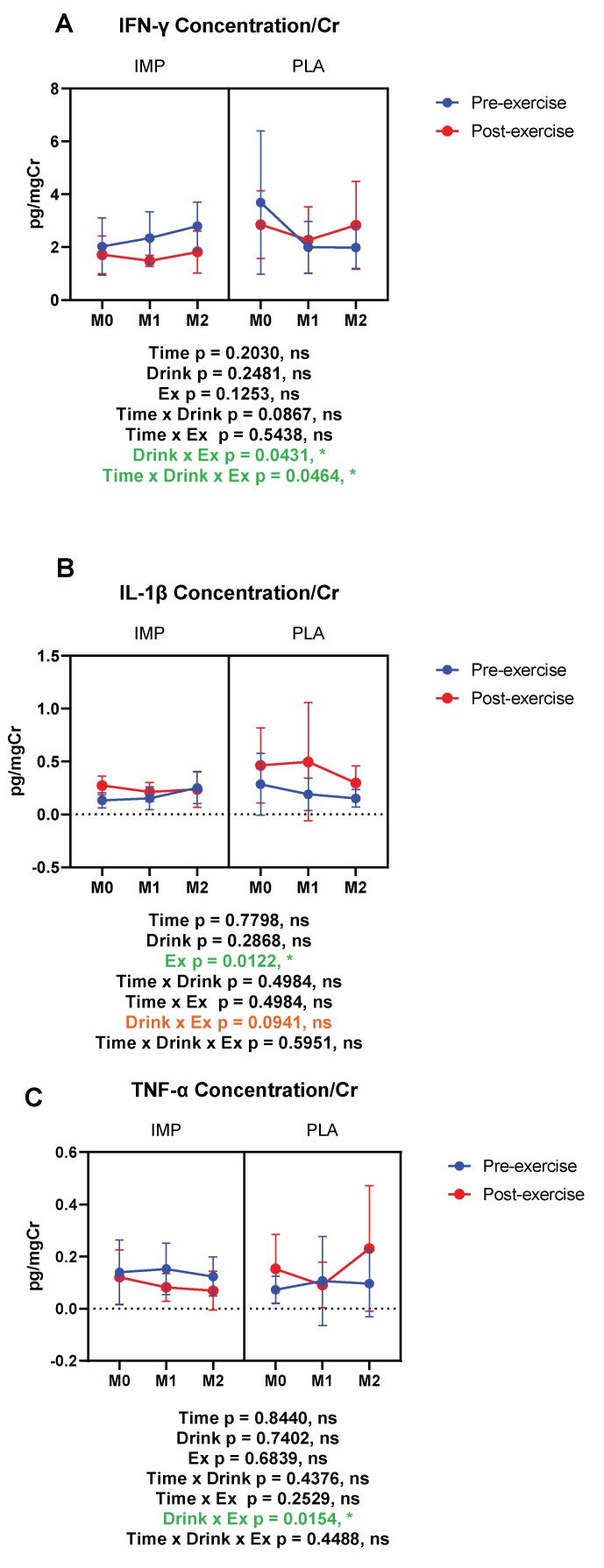
Urine interferon (IFN)-γ (**A**), interleukin (IL)-1β (**B**), and tumor necrosis factor (TNF)-α (**C**) concentrations corrected with creatinine. The data are presented as means ± SD. * p <0.05. Green text indicates a significance <0.05 and orange text indicates a significance <0.10.

The inflammatory markers IFN-γ, IL-1β, TNF-α, C5a, calprotectin, fractalkine, MCP-1, MPO, and M-CSF were measured in subjects’ urine. In the placebo period, IFN-γ, IL-1β, and TNF-α tended to increase after exercise. However, in the IMP supplementation period, the exercise-induced increase of the above cytokines was inhibited (IFN-γ and TNF-α), or trended towards being inhibited (IL-1β), according to interactions between drink and exercise (Figure 7). Summary data are available in Table 1. 

**Figure 8:**
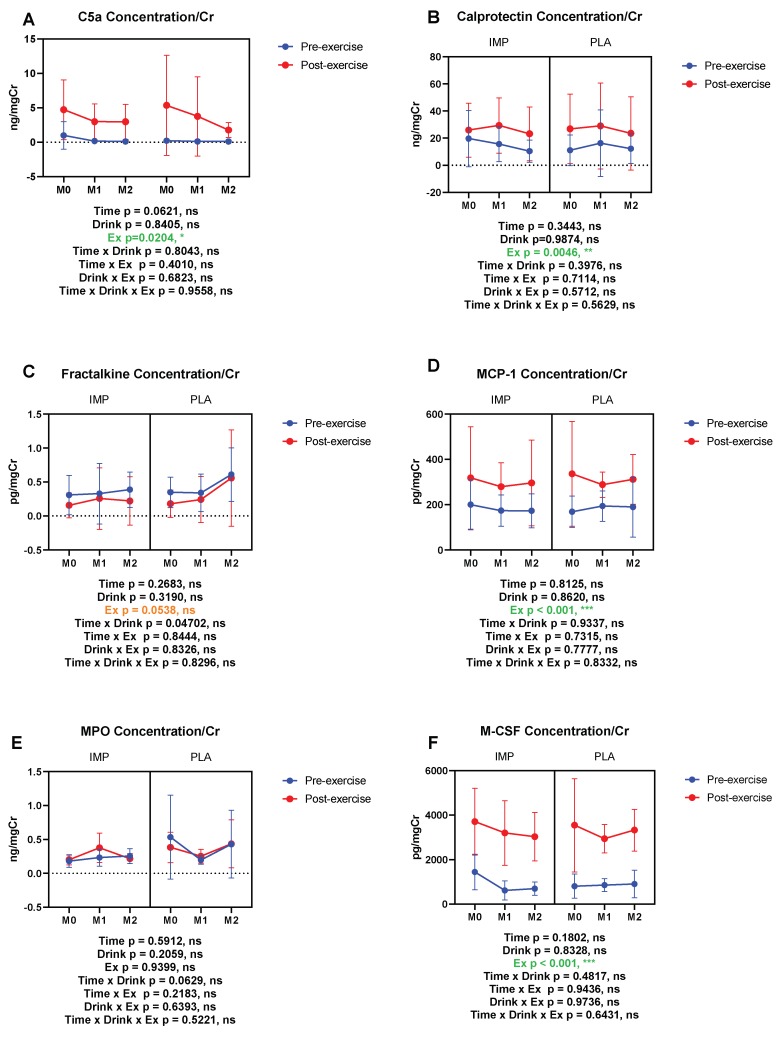
Urine complement 5a (C5a) (**A**), calprotectin (**B**), fractalkine (**C**), monocyte chemotactic protein (MCP)-1 (**D**), myeloperoxidase (MPO) (**E**), and macrophage colony-stimulating factor (M-CSF) (**F**) concentrations corrected with creatinine. The data are presented as means ± SD. * p < 0.05 and *** p < 0.001. Green text indicates a significance <0.05 and orange text indicates a significance <0.10.

C5a, calprotectin, MCP-1, and M-CSF were increased by 3000 m TTs. Fractalkine showed a trend of being decreased with 3000 m TTs, whereas urine MPO was not altered. IMP did not alter the above inflammatory substances (Figure 8). Summary data are available in Table 1. 

**Figure 9:**
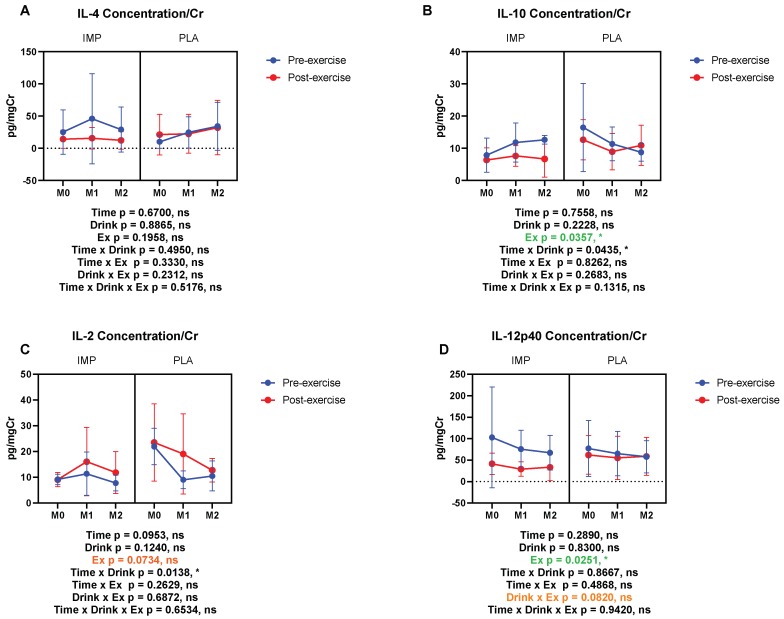
Urine IL-4 (**A**), IL-10 (**B**), IL-2 (**C**), and IL-12p40 (**D**) concentrations corrected with creatinine. The data are presented as means ± SD. * p < 0.05. Green text indicates a significance < 0.05 and orange text indicates a significance <0.10.

The concentrations of immunoregulatory cytokines, IL-2, IL-4, IL-10, and IL-12p40 were measured. Urine IL-4 was not altered by 3000 m TTs (Figure 9A). Urine IL-10 and IL-12p40 were decreased by 3000 m TTs (Figure 9B,D). Urine IL-2 showed a trend of being decreased by 3000 m TTs (Figure 9C). IMP did not alter the concentrations of the above immunoregulatory cytokines (Figure 9). Summary data are available in Table 1. 

**Figure 10:**
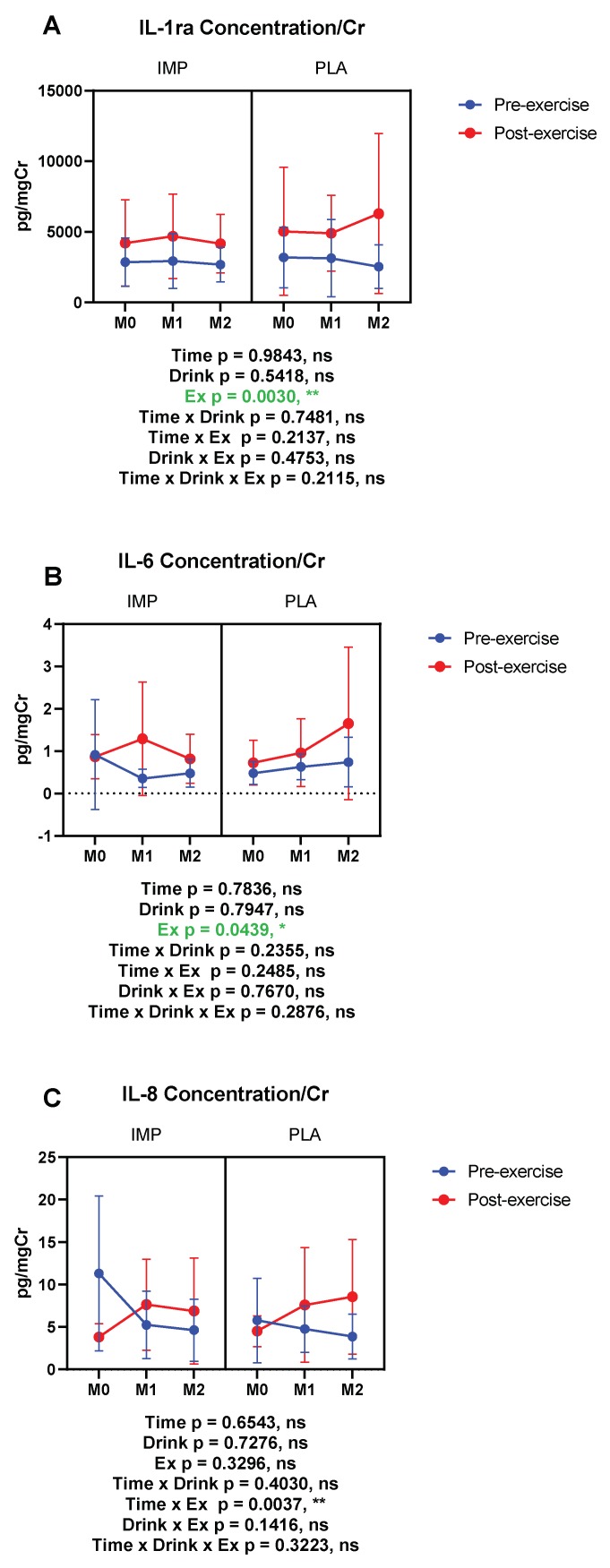
Urine IL-1 receptor antagonist (ra) (**A**), IL-6 (**B**), and IL-8 (**C**) concentrations corrected with creatinine. The data are presented as means ± SD. * p < 0.05 and ** p < 0.01. Green text indicates a significance < 0.05.

The concentrations of IL-1ra and IL-6, anti-inflammatory and multi-functional cytokines respectively, and IL-8, a neutrophil-activating cytokine, were measured. IL-1ra and IL-6 were increased by 3000 m TTs (Figure 10A,B). IL-8 was not altered by 3000 m TTs. IMP did not alter the concentrations of these cytokines (Figure 10). Summary data are available in Table 1. 

## 4. Discussion

In the present study, we used urine samples, rather than plasma or serum samples, as a non-invasive way to evaluate exercise-induced biomarker kinetics. In urine, the concentrations of C5a, calprotectin, MCP-1, M-CSF, IL-1ra, IL-6, IL-10, and IL-12p40 showed significant changes with a 3000 m TT, whereas the concentrations of fractalkine and IL-2 showed marginally significant changes. However, MPO, IL-4, IL-8, and titin were not altered in urine samples with a 3000 m TT.

Strenuous exercise may induce renal dysfunction, or even acute renal failure [33]. In the present study, an increase in the concentration of NAG indicated that a 3000 m TT induced renal tubular damage. Though IMP did not affect NAG, with 8 weeks of IMP supplementation, urine osmotic pressure and specific gravity were not significantly affected by exercise, indicating that IMP may have protective effects on renal condensing function.

Cytokine kinetics are well documented for their quick responses to strenuous exercise, reflecting a transient perturbation to the immune system [1,2,3]. We demonstrated that IFN-γ, IL-1β, and TNF-α were reduced by an 8-week IMP supplementation regimen, indicating that IMP may have potential in preventing strenuous exercise-induced inflammation. A great body of literature has demonstrated that strenuous exercise causes an inflammatory response and structural damage to the body, and that nutritional approaches have been used to prevent exercise-induced inflammatory response [1,2,3]. For example, supplementation with polyphenols and flavonoids has been used to prevent inflammation in exercise [34,35]. The potential mechanisms of this protection are thought to be related to classical inflammation pathways and the Toll-like receptor 4-mediated pathway by down-regulating down-stream protein and cytokine production [36]. Additionally, a low carbohydrate, high fat ketogenic diet has also been reported to have the potential to prevent exercise-induced inflammation [37]. Volunteers consuming a proprietary milk protein supplement for 8 weeks reported significant alleviation in joint pain and walked a significantly longer distance during a 6-min walking test [38]. Therefore, nutritional intervention may be a feasible method of protecting athletes from exercise-induced inflammation and muscle or organ damage.

Athletes report GI symptoms during training and competition frequently [39]. I-FABP is reported to reflect functional changes in exercise-induced intestinal permeability. Although the mechanism of exercise-induced GI symptoms is not fully understood, 90 min of running at a challenging pace may induce significant elevation of serum I-FABP concentrations, and symptomatic athletes have been reported to exhibit higher lipopolysaccharide (LPS) activity, indicating intestinal damage and increased intestinal permeability [39]. Although LPS from the portal circulation will be scavenged and removed from the body by Kupffer cells, LPS clearance might be overwhelmed during prolonged intense exercise, leading to the leakage of LPS from the liver into the central circulation, therefore leading to endotoxemia and exercise-induced heat stroke [40]. Several supplementation methods, including the administration of probiotics, have been considered for GI treatment in athletes. It has been reported that a carbohydrate (CHO)-containing beverage has protective effects on gastroduodenal function but showed no protective effects on intestinal function [41]. Acute oral glutamine supplementation prior to exercise prevented the rise of plasma endotoxin and nuclear factor-κB (NF-κB) activation in peripheral blood mononuclear cells [42]. However, adding glutamine to a CHO-containing beverage has no additional protective effects [43]. Fish protein hydrolysates, combined with indomethacin supplementation, reduced intestinal permeability by 62% [44]. Additionally, 4 weeks of supplementation with a multi-strain probiotic was reported to provide a small reduction (*d* = 0.25) in symptoms of gastrointestinal discomfort and increase running time to exhaustion, but failed to adjust exercise-induced plasma IL-1ra, IL-6, and IL-10 alternation [45]. According to our results, IMP supplementation significantly alleviated the elevation of I-FABP, which is a biomarker of GI permeability, benefitting GI integrity and potentially contributing to the prevention of exercise-induced endotoxemia and heat stroke.

Exhaustive exercise elicits systematic inflammatory responses and hypercytokinemia (also known as “cytokine storm”) [46]. In fact, many studies have consistently shown that interleukins, such as IL-1β, IL-1ra, and IL-6, and cytokines from the interferon family and tumor necrosis factor family increase markedly after endurance exercise [47,48,49,50]. In the present study, a 2-month IMP supplementation effectively inhibited the elevation of IFN-γ, IL-1β, and TNF-α. These preventive effects may be attributed to the protective role of IMP on LPS leakage by improving GI integrity.

Products from hyperimmunized animals have been reported for their properties in treating endogenous infections, including intestinal bacteria stimulation and rotavirus diarrhea [51]. The anti-inflammatory properties of these products have also been reported [21,24,25]. After administration of 10 g of bovine hyperimmune colostrum immunoglobulin three times a day for 5 days, healthy male volunteers showed a trend toward less diarrhea after being challenged with *Cryptosporidium parvum* [52]. The mechanism of how products from hyperimmunized animals protect individuals from endogenous infection is unknown; however, results of the present study indicate that these mechanisms may be related to the retention of GI integrity and the improvement of LPS-induced endogenous inflammation.

Similar to IMP, bovine colostrum, the “early milk,” is abundant in bioactive components, including immune, growth, and antimicrobial factors [53,54]. In a cross-over study, after 2 weeks of daily supplementation with bovine colostrum, treadmill running-induced intestinal permeability was reduced by 80% [55]. Moreover, Playford et al. reported that bovine colostrum supplementation may have the potential to reduce NSAID-induced increases in intestinal permeability [55]. The mechanisms for this action have been demonstrated as being improved maintenance of tight junctions under thermal, and possibly oxidative, stresses [52,53,54,55]. An et al. showed that bovine colostrum significantly inhibited IL-1β-induced IL-8 and intracellular adhesion molecule-1 mRNA expression, suppressed IL-1β-induced NF-κB activation and cyclooxygenase-2 protein expression levels, and blocked translocation of p65 into the nucleus in HT29 cells [56]. Bovine colostrum has been also evaluated for its antibacterial activity against *Escherichia coli*, *Staphylococcus aureus*, *Proteus vulgaris*, *Enterobacter aerogenes*, and *Salmonella typhi* [57]. Due to its similarities to colostrum, IMP might function through similar mechanisms. However, the mechanisms underlying the beneficial effects of IMP should be investigated in further studies.

In the present study, we used a 1-month wash-out period to avoid carry-over effects. This period was chosen on the basis of subjects’ activity levels and according to previous studies [28,29,30]. However, a longer wash-out period may better prevent carry-over effects. Another limitation in the present study is the lack of exercise-induced symptoms observed and reported by the participants according to the self-report questionnaires, though biochemical changes were seen with urine analyses. Whether IMP will contribute its benefits to more strenuous exercise requires validation, and further studies are encouraged.

## 5. Conclusions

We demonstrated that urine osmotic pressure, urine specific gravity, I-FABP, IFN-γ, IL-1β, and TNF-α were reduced in runners provided an 8-week IMP supplementation regimen, indicating that IMP may have the potential to prevent strenuous exercise-induced renal dysfunction, increased intestinal permeability, and inflammation. Thus, IMP supplementation may provide a feasible nutritional approach for the prevention of unfavorable exercise-induced disorders.

## Figures and Tables

**Table 1:** Summary of data from Figure 4, Figure 5, Figure 6, Figure 7, Figure 8, Figure 9 and Figure 10.

Name	Trial	M0		M1		M2	
		IMP	PLA	IMP	PLA	IMP	PLA
NAG> (IU/L)	Pre Post	2.729 ± 1.438 8.543 ± 2.540	1.714 ± 1.182 7.257 ± 4.712	2.729 ± 0.991 10.457 ± 5.369	2.943 ± 1.258 5.629 ± 1.847	2.500 ± 1.956 11.971 ± 8.136	2.471 ± 1.423 9.029 ± 7.692
UOP mOSm/L	Pre Post	878.7 ± 205.1 785.8 ± 130.2	642.5 ± 275.9 580.3 ± 179.7	869.4 ± 284.2 867.3 ± 86.36	906.2 ± 193.6 676.0 ± 169.4	672.7 ± 205.5 729.0 ± 63.38	879.7 ± 188.6 670.7 ± 211.8
SPG	Pre Post	1.026 ± 0.006 1.024 ± 0.003	1.019 ± 0.008 1.018 ± 0.006	1.025 ± 0.008 1.027 ± 0.004	1.025 ± 0.006 1.018 ± 0.009	1.019 ± 0.004 1.022 ± 0.003	1.024 ± 0.006 1.020 ± 0.006
I-FABP (pg/mgCr)	Pre Post	196.8 ± 52.90 4783 ± 2491	354.0 ± 473.5 6335 ± 2411	183.7 ± 195.1 3635 ± 1249	232.0 ± 283.4 5139 ± 2331	370.0 ± 450.9 4845 ± 2402	205.9 ± 206.8 7609 ± 4507
Titin (pg/mgCr)	Pre Post	912.0 ± 613.6 978.1 ± 637.1	1329 ± 1147 1466 ± 846.7	1050 ± 1146 802.1 ± 400.1	968.5 ± 849.9 11901 ± 720.4	828.6 ± 561.3 848.5 ± 586.2	838.8 ± 621.9 1355 ± 1238
IFN-γ (pg/mgCr)	Pre Post	2.030 ± 1.089 1.716 ± 0.710	3.694 ± 2.713 2.273 ± 1.261	2.351 ± 0.998 1.488 ± 0.206	2.004 ± 0.971 2.273 ± 1.261	2.801 ± 0.908 1.821 ± 0.797	1.990 ± 0.785 2.834 ± 1.665
IL-1β (pg/mgCr)	Pre Post	0.132 ± 0.073 0.272 ± 0.089	0.285 ± 0.292 0.462 ± 0.355	0.152 ± 0.107 0.210 ± 0.088	0.189 ± 0.153 0.496 ± 0.559	0.251 ± 0.148 0.235 ± 0.169	0.150 ± 0.084 0.297 ± 0.161
TNF-α (pg/mgCr)	Pre Post	0.139 ± 0.123 0.121 ± 0.104	0.073 ± 0.051 0.153 ± 0.132	0.153 ± 0.098 0.082 ± 0.053	0.107 ± 0.170 0.091 ± 0.087	0.124 ± 0.074 0.069 ± 0.074	0.096 ± 0.127 0/231 ± 0.240
C5a (ng/mgCr)	Pre Post	0.991 ± 1.998 4.726 ± 4.349	0.232 ± 0.219 5.362 ± 7.272	0.181 ± 0.079 2.992 ± 2.598	0.125 ± 0.068 3.766 ± 5.757	0.125 ± 0.058 2.967 ± 2.559	0.125 ± 0.057 1.782 ± 1.073
Calprotectin (ng/mgCr)	Pre Post	21.18 ± 22.26 27.31 ± 21.49	12.25 ± 11.94 29.43 ± 27.06	15.47 ± 14.25 25.68 ± 19.60	17.19 ± 26.81 31.23 ± 34.14	10.40 ± 9.010 23.56 ± 21.63	13.30 ± 11.63 26.53 ± 29.17
Fractalkine (pg/mgCr)	Pre Post	0.241 ± 0.251 0.104 ± 0.200	0.398 ± 0.200 0.276 ± 0.357	0.329 ± 0.488 0.295 ± 0.484	0.385 ± 0.274 2.276 ± 0.357	0.323 ± 0.214 0.252 ± 0.380	0.579 ± 0.422 0.356 ± 0.507
MCP-1 (pg/mgCr)	Pre Post	175.6 ± 96.93 341.7 ± 238.7	166.4 ± 75.83 364.7 ± 241.2	151.8 ± 40.74 257.2 ± 97.94	208.3 ± 60.27 294.3 ± 58.86	183.8 ± 75.61 320.48 ± 195.4	202.8 ± 139.8 325.4 ± 113.7
MPO (ng/mgCr)	Pre Post	0.181 ± 0.090 0.202 ± 0.076	0.535 ± 0.620 0.384 ± 0.224	0.234 ± 0.126 0.377 ± 0.216	0.199 ± 0.063 0.254 ± 0.102	0.256 ± 0.109 0.219 ± 0.075	0.432 ± 0.500 0.436 ± 0.354
M-CSF (pg/mgCr)	Pre Post	1206 ± 501.5 3904 ± 1550	831.9 ± 596.4 3766 ± 2213	706.1 ± 395.9 3100 ± 1563	875.2 ± 311.3 2987 ± 688.3	714.5 ± 324.1 3274 ± 977.6	714.8 ± 389.8 3375 ± 1041
IL-4 (pg/mgCr)	Pre Post	12.76 ± 17.64 14.78 ± 15.49	5.900 ± 3.816 21.63 ± 24.64	20.34 ± 18.00 10.50 ± 9.960	24.65 ± 26.71 23.54 ± 32.92	17.95 ± 19.85 10.87 ± 14.31	30.44 ± 39.17 36.49 ± 44.67
IL-10 (pg/mgCr)	Pre Post	7.875 ± 5.324 6.883 ± 3.913	18.55 ± 13.73 13.31 ± 6.581	11.12 ± 6.290 8.265 ± 3.075	11.46 ± 5.722 9.332 ± 6.126	12.88 ± 1.372 7.371 ± 5.916	9.478 ± 2.233 12.28 ± 5.593
IL-2 (pg/mgCr)	Pre Post	9.136 ± 2.035 9.079 ± 2.721	21.94 ± 7.037 23.51 ± 15.03	11.34 ± 8.41 16.08 ± 13.32	9.022 ± 3.647 19.07 ± 15.58	7.782 ± 3.061 11.80 ± 8.148	10.49 ± 5.818 12.74 ± 4.582
IL-12p40 (pg/mgCr)	Pre Post	72.08 ± 92.78 41.16 ± 27.32	82.30 ± 69.99 66.71 ± 47.09	61.79 ± 26.67 24.63 ± 13.15	64.49 ± 56.61 53.56 ± 55.21	56.42 ± 31.53 35.61 ± 33.74	49.23 ± 33.20 59.48 ± 48.20
IL-1ra (pg/mgCr)	Pre Post	2660 ± 1798 4340 ± 3328	3221 ± 2392 5053 ± 4958	2382 ± 1412 4522 ± 3244	3314 ± 2953 4690 ± 2889	2501 ± 1237 4172 ± 2276	2262 ± 1490 4211 ± 2819
IL-6 (pg/mgCr)	Pre Post	0.445 ± 0.392 0.798 ± 0.530	0.527 ± 0.267 0.780 ± 0.557	0.397 ± 0.212 1.418 ± 1.423	0.676 ± 0.302 1.021 ± 0.861	0.484 ± 0.359 0.907 ± 0.579	0.794 ± 0.619 1.839 ± 1.897
IL-8 (pg/mgCr)	Pre Post	9.168 ± 7.782 3.786 ± 1.791	6.491 ± 5.077 4.969 ± 1.629	5.460 ± 4.316 8.400 ± 5.455	5.112 ± 2.853 8.615 ± 6.825	4.541 ± 3.988 7.604 ± 6.530	3.935 ± 2.887 9.366 ± 7.042

IMP: results of immune protein trial, PLA: results of placebo trial, UOP: urinary osmotic pressure, and SPG: urine-specific gravity. The data are presented as means ± SD.
